# Raman lasing and soliton mode-locking in lithium niobate microresonators

**DOI:** 10.1038/s41377-020-0246-7

**Published:** 2020-01-20

**Authors:** Mengjie Yu, Yoshitomo Okawachi, Rebecca Cheng, Cheng Wang, Mian Zhang, Alexander L. Gaeta, Marko Lončar

**Affiliations:** 1 Department John A. Paulson School of Engineering and Applied Sciences, Harvard University, Cambridge, MA 02138 USA; 2 Department of Applied Physics and Applied Mathematics, Columbia University, New York, NY 10027 USA; 3 Department of Electronic Engineering & State Key Laboratory of Terahertz and Millimeter Waves, City University of Hong Kong, Kowloon, Hong Kong China; 4 HyperLight Corporation, 501 Massachusetts Avenue, Cambridge, MA 02139 USA

**Keywords:** Integrated optics, Microresonators

## Abstract

The recent advancement in lithium-niobite-on-insulator (LNOI) technology is opening up new opportunities in optoelectronics, as devices with better performance, lower power consumption and a smaller footprint can be realised due to the high optical confinement in the structures. The LNOI platform offers both large *χ*^(2)^ and *χ*^(3)^ nonlinearities along with the power of dispersion engineering, enabling brand new nonlinear photonic devices and applications for the next generation of integrated photonic circuits. However, Raman scattering and its interaction with other nonlinear processes have not been extensively studied in dispersion-engineered LNOI nanodevices. In this work, we characterise the Raman radiation spectra in a monolithic lithium niobate (LN) microresonator via selective excitation of Raman-active phonon modes. The dominant mode for the Raman oscillation is observed in the backward direction for a continuous-wave pump threshold power of 20 mW with a high differential quantum efficiency of 46%. We explore the effects of Raman scattering on Kerr optical frequency comb generation. We achieve mode-locked states in an X-cut LNOI chip through sufficient suppression of the Raman effect via cavity geometry control. Our analysis of the Raman effect provides guidance for the development of future chip-based photonic devices on the LNOI platform.

## Lithium niobate on insulator: the Raman effect

Better understanding, and thus control, of lithium niobate interactions with light could guide the development of an optical device that measures time even more precisely than atomic clocks. Marko Lončar of Harvard University and colleagues studied how light scatters when laser light is pumped into an optical cavity made from lithium niobate, a synthetic crystal widely used in optical materials. Their findings suggested that changing the shape of the lithium niobate cavity could help them suppress ‘Raman scattering’, a type of energy transfer that happens when light interacts with the material’s molecules. When laser light was shone through the specially tuned cavity, it exited in the form of light pulses of extremely short duration. The findings could guide the development of optical devices that can more precisely measure standard units such as distance and time.

## Introduction

The monolithic lithium-niobite-on-insulator (LNOI) platform has attracted significant interest for the realisation of next-generation nonlinear photonic devices and the observation of new nonlinear dynamics due to its large *χ*^(2)^ (*r*_33_ = 3 × 10^−11^ m/V) and *χ*^(3)^ nonlinearities (*n*_2_ = 1.8 × 10^−19^ m^2^/W)^1–16^. The LNOI platform is creating new opportunities for large-scale integration of optical and electronic devices on a single chip, as it combines the material properties of lithium niobate with the integration power of nanophotonics. By leveraging recent advances in the fabrication of ultra-low-loss lithium niobate (LN) nanowaveguides and microring resonators^1^, researchers have demonstrated Kerr optical frequency combs (OFCs)^2–4^, broadband electro-optic combs^5^, highly efficient second harmonic generation^6–8^ and multiple-octave-spanning supercontinuum generation (SCG)^8,9^. LN is known as a Raman-active crystalline material with several strong vibrational phonon branches in different polarisation configurations^17–23^. There has been evidence of Raman scattering in LN discs or whispering gallery resonators^24,25^ fabricated by mechanical polishing^26^. The Raman effect in integrated photonic devices not only enables Raman lasers for the generation of new frequencies at low optical powers^27–34^ but can also lead to nontrivial nonlinear interactions through tailoring the dispersion properties, such as the interplay between the Raman effect, and both *χ*^(2)^ and *χ*^(3)^ effects, impacting Kerr comb formation, electro-optic comb formation and supercontinuum generation^35–41^. Recent work by Hansson et al. has shown that aligning the cavity free spectral range (FSR) to the Raman gain can allow for the generation of an octave-spanning Raman frequency comb^42^. The LN photonic circuit is particularly appealing for microresonator-based Kerr frequency comb generation, since the presence of a large second-order nonlinearity *χ*^(2)^ offers advantageous functionality for a fully on-chip optical clock and metrology, a key element missing from current mature silicon nitride or silica technologies. Due to the large Raman gain in a crystalline material, a strong interplay between Raman scattering and four-wave mixing (FWM) has been observed in materials such as diamond and silicon^36^, and strategies have been proposed to suppress these interactions by controlling the FSR^36,39,43^. However, Raman scattering and its influence on soliton mode-locking remain largely unexplored in dispersion-engineered monolithic LNOI devices.

In this paper, we demonstrate multi-wavelength Raman lasing in an X-cut high-*Q* LN microresonator with Raman frequency shifts of 250 cm^−1^, 628 cm^−1^, and 875 cm^−1^ via pumping with the transverse electric (TE) polarisation and a shift of 238 cm^−1^ with the traverse magnetic (TM) polarisation. The dominant Raman oscillation occurs in the backward direction with respect to the pump, and the backward Raman gain coefficient is measured to be 1.3 cm/GW for the 250 cm^−1^ Raman shifted line for the TE polarisation and 0.07 cm/GW for the 238 cm^−1^ Raman shifted line for the TM polarisation. In both cases, a 1.5-μm pump is used to excite the sample. To our knowledge, this is the first characterisation of multi-wavelength Raman lasing on a monolithic LN chip. In addition, we investigate the effects of the Raman process on Kerr comb generation and soliton mode-locking for both polarisations and show that the Raman effect can be controlled to enable mode-locked Kerr comb formation for the TM polarisation.

## Results

**Fig. 1:**
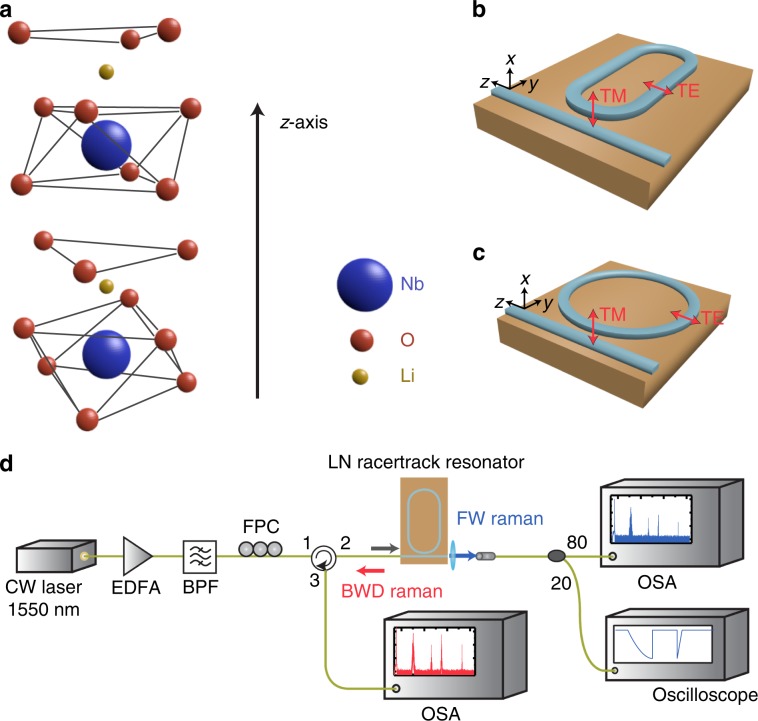
Experimental setup. **a** Schematic of the LN crystalline structure. The crystal axis is along the *z*-axis. **b**, **c** Orientation of the LN microresonator on an X-cut wafer. A 30-GHz free spectral range (FSR) racetrack resonator is used for the Raman characterisation. **b** A 250-GHz FSR microring resonator is used for the Raman–Kerr interactions. **c** The TE and TM polarisations are also indicated. **d** Setup for the forward (FW) and backward (BWD) Raman characterisation. EDFA erbium-doped fibre amplifier, BPF bandpass filter, FPC fibre polarisation controller, OSA optical spectrum analyser.

LN is a uniaxial material with its crystal axis along the z direction, as shown in Fig. 1a. LN devices are fabricated on an X-cut thin-film wafer, where the *x*-axis is normal to the wafer plane. LN has two Raman-active phonon symmetries: the A symmetry polarised along the *z*-axis and the E symmetry polarised in the degenerate *x*–*y* plane^19,21^ due to the atomic vibration. Furthermore, both transverse (TO) and longitudinal (LO) optical phonon modes exist. An X-cut wafer allows access to both TO and LO modes in both symmetries. The selection rules of Raman scattering depend on the wavevectors and polarisation of the pump and Stokes fields^17–20^. Two different cavity geometries are used in our experiments (Fig. 1b, c), where the TE-polarised light is mostly parallel to the crystal axis in the racetrack geometry and the TM-polarised light is parallel to the non-polar axis (*x*-axis).

### Characterisation of Raman scattering

**Fig. 2:**
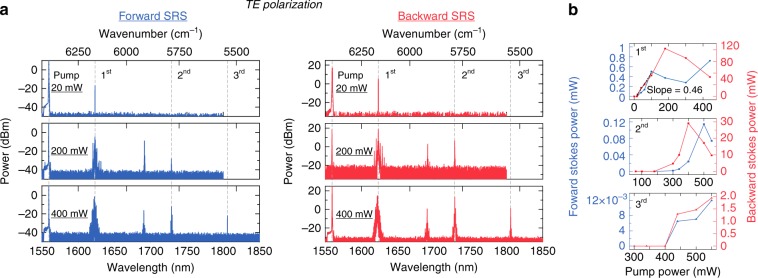
Raman emission for TE-polarised light from an X-cut LN racetrack microresonator. **a** Three Raman spectra (from top to bottom) at pump powers of 20, 200, and 400 mW, which correspond to the threshold power for the Raman oscillation with frequency shifts of 250 cm^−1^, 628 cm^−1^, and 875 cm^−1^, respectively. The Stokes and pump are TE-polarised and are along the LN polar *z*-axis. Both the forward (blue) and backward (red) spectra are shown. The cascaded Raman peak from the 1st Stokes field is also observed at 1691 nm. **b** Raman emission power (top to bottom: 1st to 3rd Stokes) as a function of the pump power. The threshold for the 1st Stokes peak in the backward direction is 20 mW with a high differential conversion efficiency of 46% (linear fit shown with the black dashed line). The deviation from linear growth above a pump power of 60 mW is due to mini-comb formation and other Raman oscillations.

**Fig. 3:**
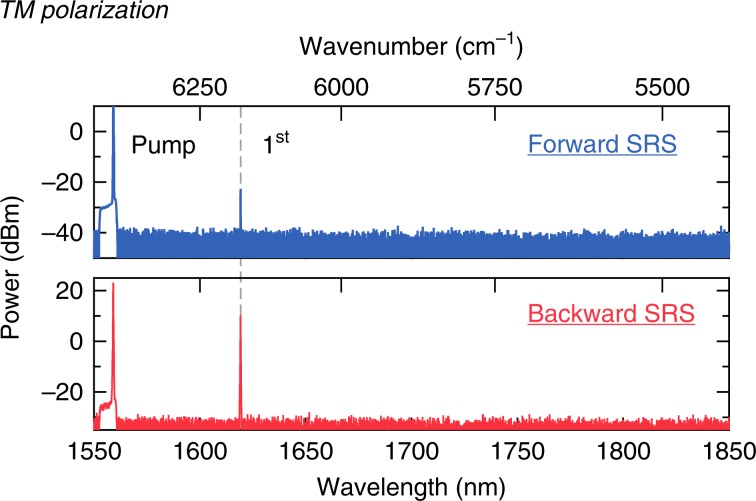
Raman emission for the TM mode (the light polarisation is along the *x*-axis) at a frequency shift of 238 cm^−1^ at the threshold power of 340 mW.

The experimental setup for Raman characterisation is shown in Fig. 1d. We inject an amplified continuous-wave (CW) pump laser centred at 1560 nm into a monolithically integrated LN racetrack microresonator [Fig. 1b]. The device is cladded with silicon oxide with a top waveguide width of 1.2 μm and an etch depth of 450 nm on an 800-nm-thick LN thin film. The racetrack design allows for two long straight waveguide regions to maximise the interaction with the TO optical phonon mode for the TE polarisation. The FSR of the microresonator is 30 GHz, which is within the Raman gain bandwidth^19^. The intrinsic *Q* of the resonator is ~1.5 × 10^6^ for both the TE and TM modes (see Supplementary Information). We record the Raman emission spectra in both the forward and backward directions using an optical circulator and two optical spectrum analysers at various pump powers in the bus waveguide. For the TE polarisation, we observe several Raman oscillations [Fig. 2a] with frequency shifts of 250 cm^−1^ (7.5 THz), 628 cm^−1^ (18.8 THz), and 875 cm^−1^ (26.2 THz), corresponding to the optical phonon branches of A(TO)_1_, A(TO)_4_, and A(LO)_4_, respectively. The 1st Stokes peak (250 cm^−1^) has the lowest pump threshold of 20 mW with a high differential conversion efficiency of 46%, as shown in Fig. 2b. To our knowledge, this is the highest quantum conversion efficiency reported in an LN material. As the pump power increases, a mini-comb starts to form around the 1st Stokes peak due to the anomalous group velocity dispersion (GVD). This mini-comb prevents the first Stokes line from monotonically increasing with power. In addition, cascaded Raman peaks form ~1691 nm at 170 mW of pump power in the forward direction. Notably, strong spectral peaks at 1691 nm are also observed in the backward direction, largely due to an FWM process where the dominant 1st Stokes line acts as the pump. The 2nd and 3rd Stokes peaks appear as the pump power reaches 200 mW and 400 mW, respectively. The efficiency of the Raman effect is higher in the backward direction, which is phase-matched^44^; this is particularly true for microscale waveguides that feature a non-negligible longitudinal electric field component^45^. This asymmetric gain can also be attributed to strong polaritonic effects that affect the phase-matching conditions in the forward direction^24,46^. We estimate the Raman gain *g*_R_ of the 1st Stokes line to be 1.3 cm/GW based on^33^. For the 2nd and 3rd Stokes peaks, we are unable to extract the Raman gain due to the presence of Kerr and other Raman processes influencing the pump power. Previously, the Raman gain of the corresponding mode in bulk LN was reported to be 12.5 cm/GW at 488 nm by Bache^23^, 8.9 cm/GW at 694 nm by Boyd^21^, and 5 cm/GW at 1047 nm by Johnson & Chunaev et al.^22^, in good agreement with our measurement based on the relation *g*_R_ ∝ (*λ*_p_ *λ*_s_)^−1^, where *λ*_p_ is the pump wavelength and *λ*_s_ is the Stokes wavelength^29^. Similarly, we characterise the devices using a TM-polarised pump, where the light polarisation is along the *x*-axis. As shown in Fig. 3, only one Raman oscillation is observed with a frequency shift of 238 cm^−1^ [E(TO)_3_] at a threshold power of 340 mW, which corresponds to a Raman gain of 0.07 cm/GW.

### Kerr comb generation and mode-locking

**Fig. 4:**
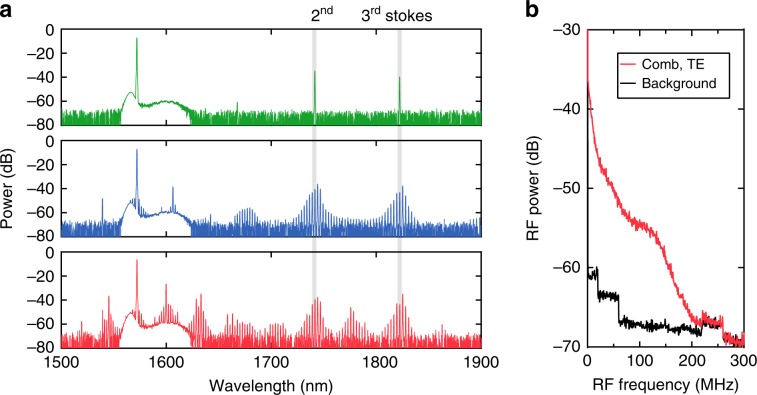
Kerr frequency comb generation for TE-polarised light. **a** Raman and Kerr oscillations for the TE mode from an LN microresonator as the pump laser is tuned into the cavity resonance (top to bottom). **b** RF spectrum corresponding to the bottom spectra in **a**.

Next, we investigate the effects of Raman scattering on Kerr comb formation for both the TE and TM polarisations. Mode-locked Kerr frequency combs are particularly attractive on an LN chip for optical metrology, since the combination of large *χ*^(2)^ and *χ*^(3)^ nonlinearities could enable direct on-chip self-referencing without external amplifiers or a periodically poled LN crystal. In order to achieve soliton mode-locking in the presence of strong Raman scattering, a microring with a smaller radius is preferred to achieve FWM oscillations (the broadband FWM gain peaks at 0.15 cm/GW)^36^. Here, we pump an air-cladded LN microring resonator with a radius of 80 µm, which corresponds to an FSR of 250 GHz (Fig. 1b). The pump power in the bus waveguide is 400 mW for both polarisations. The LN devices here are air-clad with a top waveguide width of 1.3 µm and an etch depth of 350 nm on an X-cut 600-nm-thick LN thin film. The cross-section is engineered to allow for anomalous GVD for both polarisations (see Supplementary Information). We measure a loaded *Q* of >1.5 × 10^6^ for both the TE and TM modes. Figure 4 shows the Kerr comb generation dynamics for the TE polarisation. We measure the generated spectrum as the pump is tuned into a cavity resonance. As power builds up in the cavity, we observe strong Raman peaks that correspond to the phonon branch of A(TO)_4_ and A(LO)_4_ (Fig. 4a, top), while the A(TO)_1_ mode is successfully suppressed. With further pump detuning, we observe the formation of primary sidebands, due to the parametric gain, for Kerr comb formation (Fig. 4a, middle) and mini-comb formation around the primary sidebands (Fig. 4a, bottom)^47^. The RF spectrum corresponding to the bottom state in Fig. 4a is shown in 4b, indicating that the comb is in a high-noise state. This result is largely due to the strong Raman effect that competes with the FWM interaction and prevents mode-locking^35,36^.

**Fig. 5:**
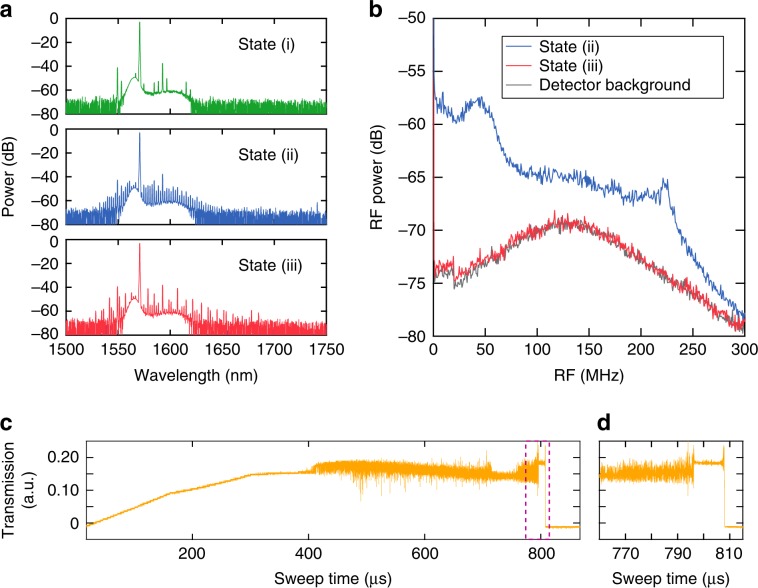
Kerr frequency comb generation for TM-polarised light. **a** Kerr oscillation for the TM mode from an X-cut LN microresonator as the pump laser is red-detuned into the cavity resonance (top to bottom). **b** RF spectra corresponding to States (ii) and (iii) in **a**. **c** Transmission measurement of the microresonator output above 1570 nm, which excludes the pump wavelength. The laser is tuned to the resonance as the wavelength increases, dominated by the thermal-optic effect. The laser sweeping speed is 80 pm/ms. **d** Zoom-in of the transmission trace in **c**, which shows the onset of the soliton step.

Figure 5 shows the comb dynamics for the TM mode. Unlike the case for the TE mode, the Raman effect is much weaker, and we do not observe a Raman oscillation at these pump powers; this is attributed to the fact that the Raman gain is less than the Kerr gain for the larger FSR. Figure 5a shows the generated spectra for an increased red-detuning of the pump. We observe the primary sidebands [State (i)], high-noise state [State (ii)], and multi-soliton state [State (iii)]. The RF spectra (Fig. 5b) show the reduction in the RF noise from State (ii) to State (iii), which strongly suggests the formation of a phase-locked state with narrow linewidth comb lines. By further detuning the pump laser, another phase-locked state with a more periodic spectral modulation is observed, which corresponds to the formation of a 5-soliton state within one cavity roundtrip (see Supplementary Information). However, a single soliton is not achieved in the passive LN device. A fast switching approach, such as electrical tuning of the cavity resonance, could be utilised to possibly overcome the instabilities resulting from both the thermal-optic and photorefractive effects. Figure 5c shows the transmission measurement of the resonator as the pump wavelength is swept through the resonance. The output is optically filtered using a long-pass filter with a cut-on wavelength of 1570 nm. Figure 5d shows a zoom-in of the dashed-rectangular region in Fig. 5c that indicates the ‘soliton step’ representing soliton formation^48^. The achieved mode-locked Kerr comb generation indicates that operating with the TM mode using an X-cut LN thin film allows for sufficient suppression of Raman effects. In contrast to the Z-cut LN thin film in ref. ^3^, where the behaviour of self-starting or bidirectional tuning is observed as a result of the photorefractive effect, our microresonator is dominated by the thermo-optic effect. Moreover, our results do not indicate the occurrence of Raman self-frequency shifting observed by ref. ^3^, which often occurs in an amorphous material. The difference in dynamics may be attributed to a thin film with different crystal orientations. We report the first demonstration of soliton mode-locking in X-cut LN microresonators compatible with active electrode control^2,5^.

## Discussion

In conclusion, we achieve multi-wavelength Raman lasing on a monolithic LN chip and characterise the distinct Raman processes for different pump polarisations. All the Raman oscillations are dominant in the backward direction with respect to the pump, and we report the highest pump-to-Stokes conversion efficiency of 46% for TE-polarised light. Operating in the normal GVD regime using a resonator with a higher *Q* will enable a highly efficient Raman laser on an LN chip. Counter-propagating pump and Stokes fields might lead to richer nonlinear dynamics or functionalities such as symmetry breaking^49^, counter-propagating solitons and Stokes solitons^50^. In addition, we observe nontrivial interactions between the Raman effect and *χ*^(3)^-based FWM processes for Kerr comb formation. Although the strong contribution from the Raman effect impedes soliton formation for the TE polarisation, we demonstrate mode-locking for the TM polarisation through an optimisation of the cavity geometry. Alternatively, TE-polarised phase-locked combs could be achieved with the help of strong electrical driving^5,41^. This work provides deep insight into the dynamics and effects of Raman scattering in the LNOI platform, which is critical for the design and development of chip-based nonlinear photonic devices.

## Materials and methods

### Device parameters and fabrication

For the Raman characterisation, we fabricate a racetrack microresonator from a commercial X-cut lithium niobate (LN) on an insulator wafer with a thin-film LN thickness of 800 nm. The device is cladded with silicon oxide 750 nm in thickness with a top waveguide width of 1.2 µm and a slab thickness of 350 nm. The bending structure is based on Euler curves to avoid mode conversion between the transverse electric (TE) and transverse magnetic (TM) modes. The two straight sections are each 1.75 µm in length along the *y*-axis. The coupling gap between the bus waveguide and microresonator is 0.75 µm, which leads to near-critical coupling for the TE modes and 45% transmission on resonance for the TM modes at 1550 nm (see Supplementary Information). The FSR is 30 GHz. For Kerr comb generation, the microring resonator is fabricated on a 600-nm-thick thin-film LN wafer with a radius of 80 µm. The device is air-cladded with a slab thickness of 250 nm and a top waveguide width of 1.3 µm. The coupling gap is 830 nm, which results in 50% transmission on resonance for the TE mode and 83% for the TM mode at 1550 nm.

Electron-beam lithography (EBL, 125 keV) is used for patterning the waveguides and microcavities in hydrogen silsesquioxane resist (FOX). Then, the patterned LN wafer is etched using Ar^+^-based reactive ion etching by a user-defined etch depth. The SiO_2_ cladding is deposited by plasma-enhanced chemical vapour deposition. Finally, the chip facet is diced and polished, which typically results in a fibre-to-chip facet coupling loss of 7 dB.

### Comb characterisation

The group velocity dispersion (GVD) is simulated using commercial finite element analysis software (COMSOL) based on the fabricated device geometry. Anomalous GVD is achieved for both the TE and TM modes at telecommunication wavelengths (see Supplementary Information). A continuous-wave pump laser (Santec TSL-510) at 1570 nm is amplified by an L-band erbium-doped fibre amplifier (EDFA, Manlight) and sent to the microring resonator using a lensed fibre after a polarisation controller. The tuning of the laser is controlled by a piezo controller. The output is collected by an aspheric objective followed by a fibre collimator. A 90:10 fibre beamsplitter is used to separate the output light into two arms. The 10% arm is sent to an optical spectrum analyser, and the 90% arm is sent through a home-built 4-*f* shaper (effectively a bandpass filter, 1575–1630 nm) to filter the pump. The filtered comb spectrum is sent to a photodetector (Newport 1811, 125 MHz) and a real-time spectrum analyser (Tektronix RSA5126A). For Fig. 5c, a functional generator with a triangular function is sent to the piezo controller to scan the laser wavelength at 70 Hz, and the filtered comb is collected by a photodetector (Thorlabs, PDA05CF2) followed by an oscilloscope (Tektronix DPO2024, 200 MHz).

## Supplementary information

Supplemental Material

## Data Availability

The data that support the plots within this paper and other findings of this study are available from the corresponding author upon reasonable request.
