# Supplementary material for: Raman lasing and soliton mode-locking in lithium niobate microresonators
Source: Light Sci Appl. 2020 Jan 20;9:9. doi: 10.1038/s41377-020-0246-7 (PMC6970987; doi:10.1038/s41377-020-0246-7)
Supplement: Supplementary file 1 — Supplemental Material [file 41377_2020_246_MOESM1_ESM.docx]

**Supplementary Information**

**Device *Q*-factor**

As shown in Fig. S1, we measure the TE resonance to be near critical coupled and the TM resonance to be over coupled with 45% transmission on resonance, both of which is extracted to have 1.5 million intrinsic *Q*-factor.

**
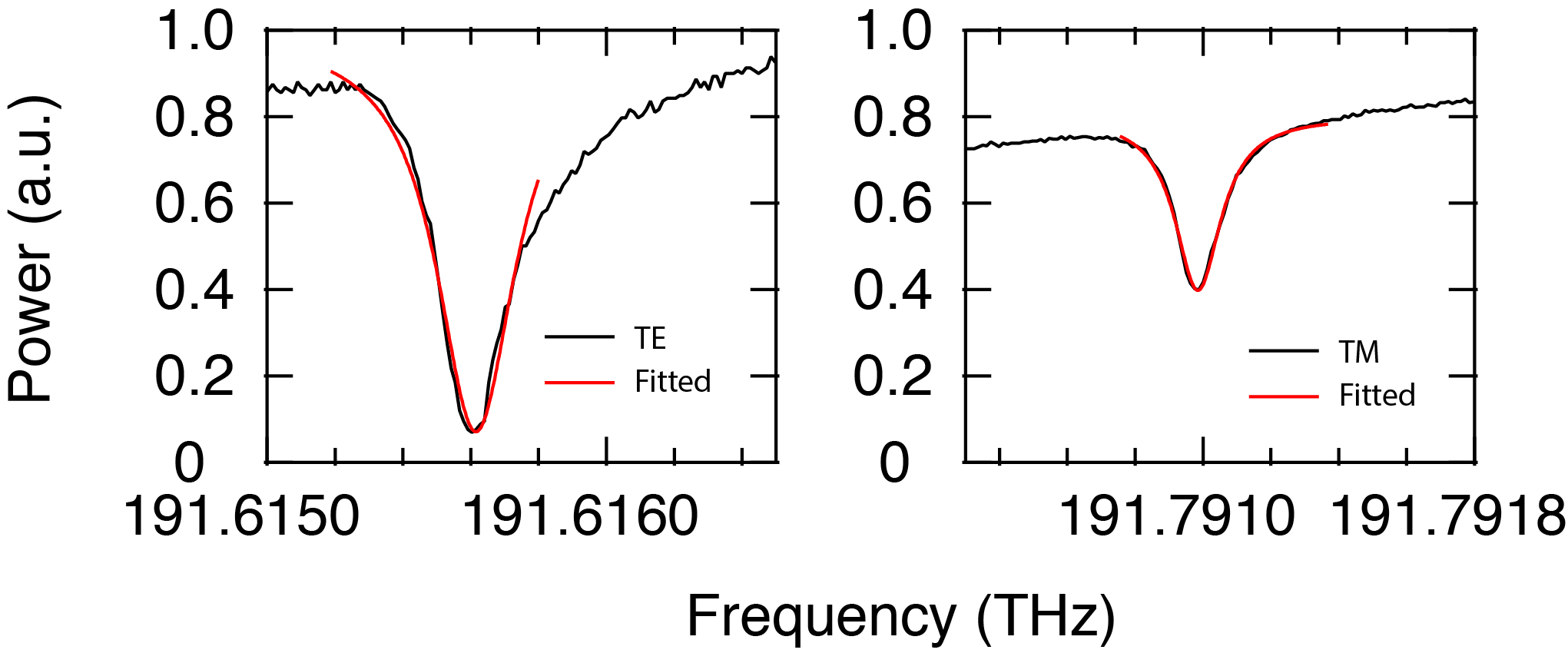
**

**Figure S1 |** The *Q*-factor measurement of the racetrack microresonator. The measurement is taken at telecommunication wavelengths for both TE and TM polarization.

**Numerical simulation of group velocity dispersion**

Figure S2 shows the cross section of fabricated LN devices which is used for modelling in COMSOL. LN is a negative uniaxial material which has ordinary and extraordinary refractive indexes (*n*_o_ and *n*_e_). The effective refractive index *n*_eff_ of the waveguide for both TE and TM polarization is simulated separately for *n*_o_ and *n*_e._ For the X-cut wafer, *n*_o_ is used for simulating the guided optical modes for TM polarization while both *n*_o_ and *n*_e_ are used for TE polarization since the TE light sees different indices as it circulates the resonator. Group velocity dispersion (GVD) is calculated based on the equation $\mathrm{GVD}=\frac{\partial^{2}k}{\partial^{2}\omega}$ where *k* is the wavenumber (= $\frac{2\pi n_{eff}}{\lambda}$) and $\omega$ is the angular frequency. Figure S3 and S4 plot the GVD curve based on the device parameters we use for Raman characterization and Kerr comb generation experiments, respectively. The GVD for the TE polarized mode is further processed using certain ratios of *n*_o_ and *n*_e_ based on the light rotation in certain microcavities. For example, 12.5 % GVD of *n*_o_ and 87.5 % GVD of n_e_ are used for racetrack microresonators, and 50 % GVD of *n*_o_ and 50 % GVD of *n*_e_ are used for microring resonators.

**
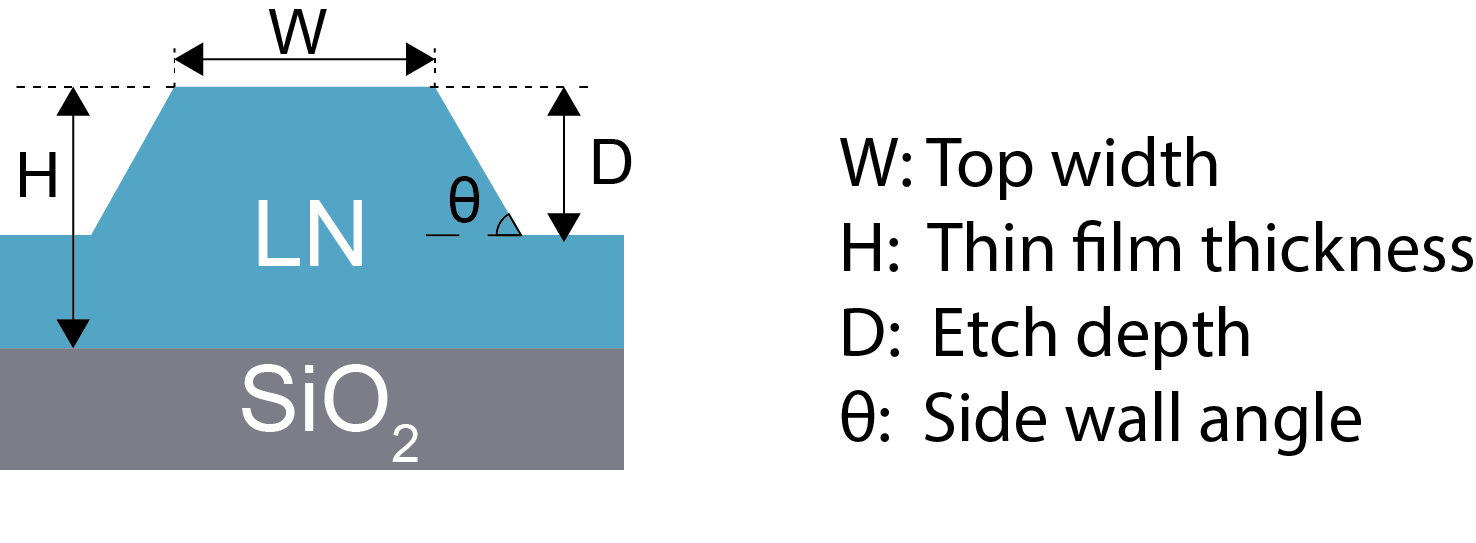
**

**Figure S2 |** The cross section of the LN device on insulator. The geometry is defined by top width (W), thin film thickness (H), etch depth (D) and sidewall angle (θ). The LN slab is due to partial etch (slab thickness = H – D).

**
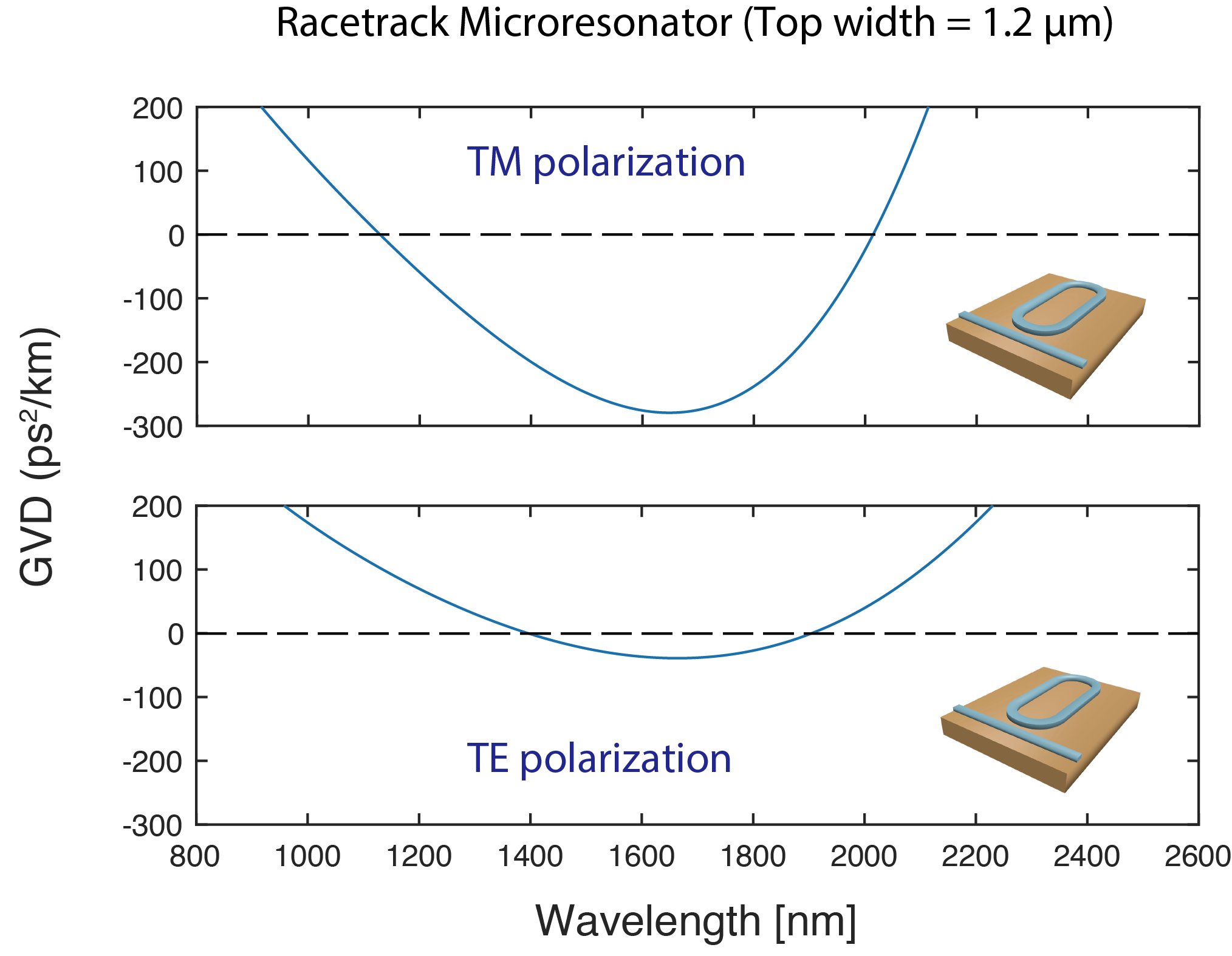
**

**Figure S3 |** The simulated group velocity dispersion for the fundamental optical mode at both TE and TM polarizations for the racetrack microresonator on a X-cut wafer.

**
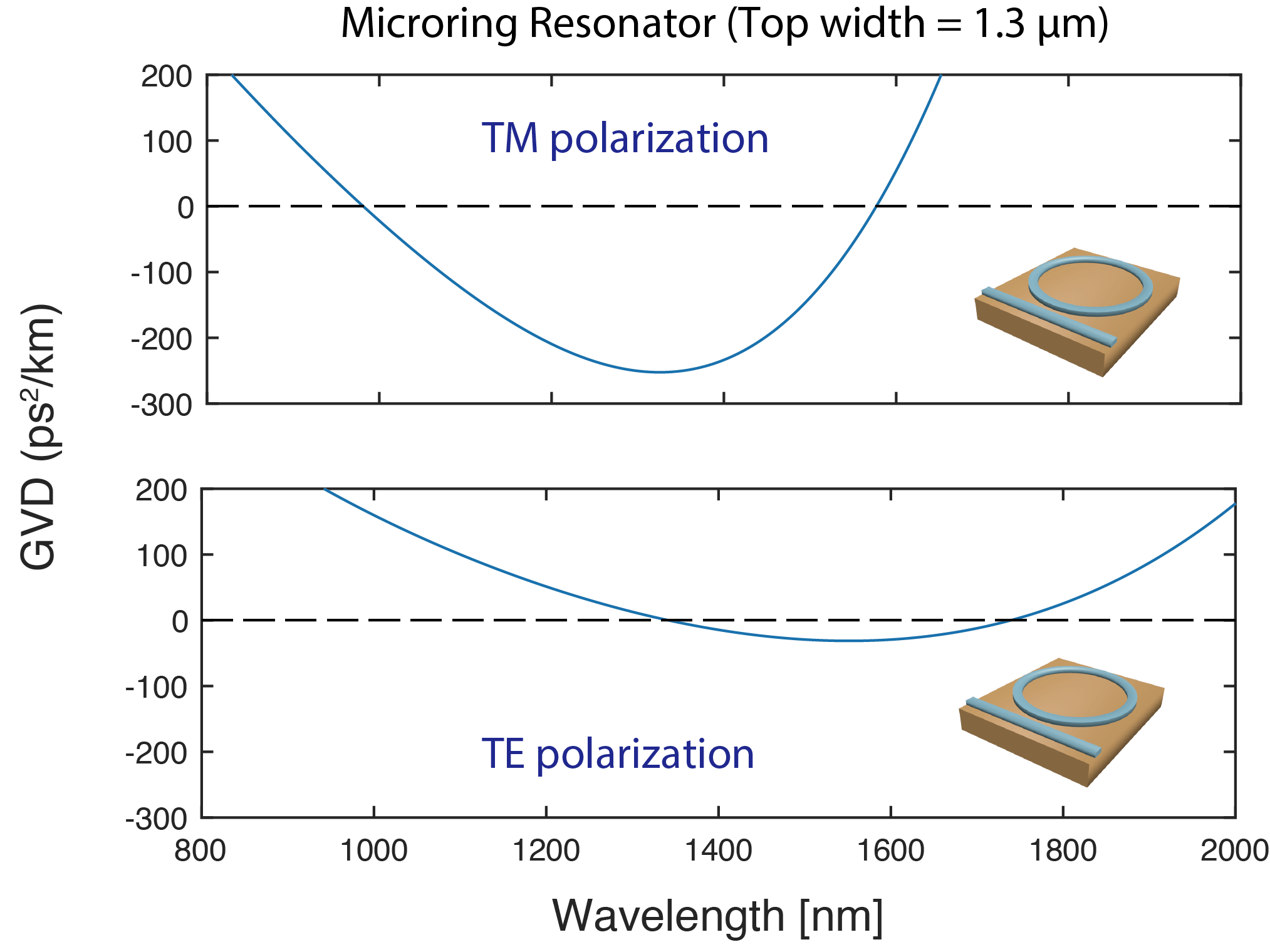
**

**Figure S4 |** The simulated group velocity dispersion for the fundamental optical mode at both TE and TM polarizations for the microring resonator on a X-cut wafer.

**Multi-soliton state**

Through small detuning of the pump laser from the low-noise State(iii) in Fig. 5, we observe another multi-soliton state where solitons are more regularly spaced in the time domain, and therefore its spectrum features a periodic modulation (red solid curve, Figure S5). By comparing the data with our numerical model, shown in Fig. S5, we estimated 5 solitons circulating inside our resonator. Each soliton has a 3-dB bandwidth of 32 nm. Comb lines are sampled at each spectral point evenly spaced at FSR (~250 GHz). Due to the fact that the cavity roundtrip time *t_rt_* is not an integer multiple of the soliton separation *t_soliton_* , comb lines are not sampled at the peak positions of the 5-soliton envelope (inset), and thus the comb spectrum appears asymmetric with respective to the pump (in this case, *t_rt_*/*t_soliton_* = 5.2). To precisely characterize the soliton state, confirm the number of solitons and their separation, auto-correlation measurement is needed to obtain the spectral phase profile or time domain information*.* In our experiment, we do not observe a single soliton state by changing pump detuning. We attribute this to the nonlinear dynamics of both thermo-optic effect and photorefractive effect in LN crystal orientation used in our experiment. This is a topic of ongoing experiments in our lab.


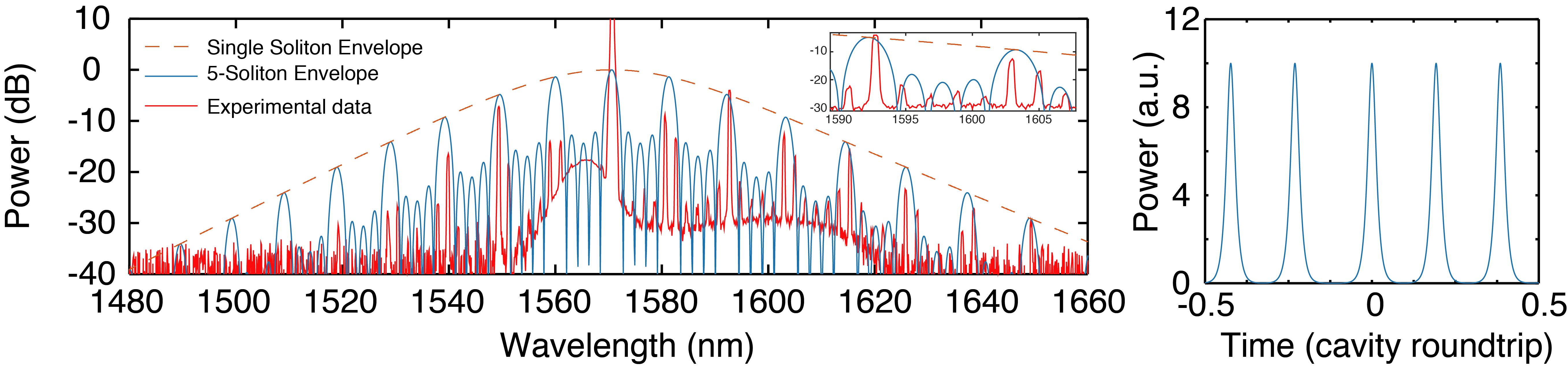


**Figure S5 |** Multi-soliton state. A different low-noise multi-soliton state (red curve, left) is achieved by detuning the pump laser from the state (iii) in Fig. 5(a). The comb spectrum is fitted with the numerical simulation corresponding to a 5-soliton spectral envelope (blue curve). A single soliton envelope is also plotted with a 3-dB spectral bandwidth of 32 nm. The time domain modeling of the 5-soliton state is shown (right). Here, *t_rt_*/*t_soliton_* = 5.2 where *t_rt_* is the cavity roundtrip time and *t_soliton_* is the soliton separation. Due to the non-integer property of *t_rt_/t_soliton_*_,_ the comb lines are not at the local peak positions of the simulated 5-soliton envelope (inset).
